# Distal tibia osteochondral allograft as a successful treatment for a glenoid chondral defect in a pediatric patient

**DOI:** 10.1016/j.xrrt.2024.02.005

**Published:** 2024-03-16

**Authors:** Logan Radtke, Cameron Guy, Adrik Da Silva, Travis Maak, Peter Chalmers

**Affiliations:** Department of Orthopaedics, University of Utah, Salt Lake City, UT, USA

**Keywords:** Pediatrics, Glenoid osteochondral defect, Distal tibia allograft, Shoulder, Microfracture, Return to sport

Osteochondral defects (OCDs) of the glenohumeral joint have been described in both the pediatric and the adult population, though their prevalence remains uncertain due to the limited literature on this subject. Some reports suggest that chondral defects of the glenohumeral joint are identified anywhere from 5% to 17% of diagnostic procedures with a higher occurrence in overhead athletes.^5^^,^^7^^,^^17^ It is postulated that the increasing participation of young athletes in competitive sports may contribute to a rising incidence of this pathology in the pediatric population.^6^ Etiologies for OCDs encompass a range of possibilities, including repetitive microtrauma, degenerative progression, postsurgical chondrolysis, vascular insult, inflammatory arthropathy, joint instability, rotator cuff damage, osteochondritis dissecans, infection, and idiopathic causes.^5^^,^^7^^,^^21^^,^^22^

OCDs of the shoulder typically present with nonspecific complaints including deep-seated shoulder pain, generalized shoulder achiness exacerbated by physical activity, acute pain following traumatic insult, crepitation with range of motion, and even shoulder instability with the potential for loss of chondrolabral containment.^5^^,^^10^^,^^16^^,^^24^ While there is literature supporting the accelerated progression of osteoarthritis in patients with untreated OCD lesions, the optimal approach to treating glenoid OCD lesions remains a subject of active research.^4^^,^^5^^,^^7^^,^^17^

This case report describes the successful two-year outcome of a distal tibial allograft transplantation for the treatment of an OCD of the glenoid in a highly active pediatric gymnast who had previously failed extensive nonoperative management and microfracture.

## Case

The patient is an otherwise healthy young female who originally presented at age 16 with the chief complaint of right shoulder pain. Her pain began years prior with an insidious onset during gymnastics. Physical exam did not indicate any signs of instability on provocative testing and there were no signs of rotator cuff injury. The only pertinent positive exam finding was tenderness over her biceps tendon. Nonoperative management including a cortisone injection, one year of physical therapy, and rest did not alleviate the patient’s pain and thus a magnetic resonance image (MRI) was obtained. MRI of the patient’s right shoulder revealed evidence of a tear of the long head of the biceps tendon as well as an OCD in the superior margin of the glenoid.

**Figure 1:**
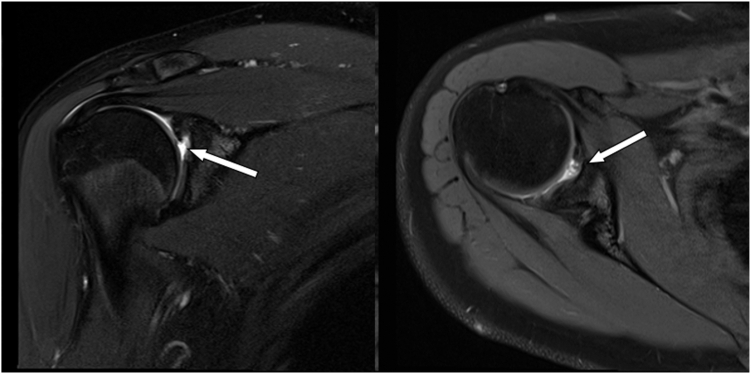
Four months post-microfracture coronal (*left*) and axial (*right*) T2-weighted MRI of right shoulder demonstrating evidence of osteochondral defect (measuring roughly 7 × 9 mm) at the anterosuperior margin of the glenoid. *MRI*, magnetic resonance image.

The patient underwent an arthroscopic microfracture, biceps tenodesis, and extensive débridement guided by intraoperative findings. At the patient’s four-month postoperative visit, she denied improvement in shoulder pain and thus another MRI was obtained, which revealed a persistent 7 × 9 mm anterosuperior OCD in the glenoid without evidence of healing following microfracture as depicted in Figure 1. The patient and her family consented to osteochondral allograft (OCA) of the glenoid defect, which was performed by the 2 senior authors of this article.

**Figure 2:**
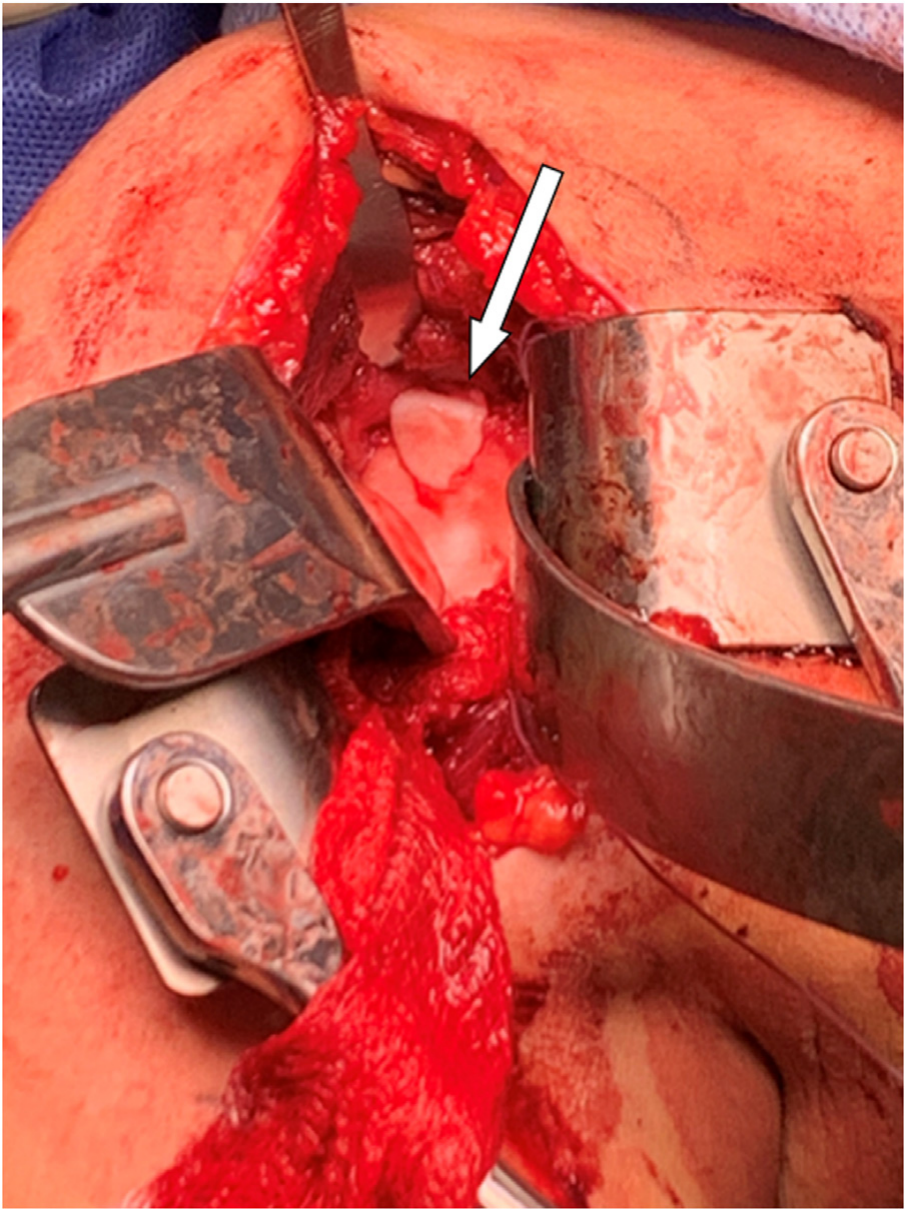
Intraoperative photo demonstrating the placement of the OCA plug within the glenoid. Clinical assessment revealed a stable construct with flush contour and well-aligned margins. *OCA*, osteochondral allograft.

This procedure was performed in an open manner using the standard deltopectoral approach in the beach chair position. Visualization of the glenoid defect was inadequate through the rotator interval and thus a minimalized subscapularis tenotomy was performed. Being mindful of the supraspinatus insertion, anterior and superior glenoid retractors were used to obtain proper visualization of the glenoid defect. Intraoperatively the OCD measured 8x10 mm along the superior glenoid. A central pin was placed with approximately 20 degrees of posterior and 5 degrees of inferior angulation and used to ream a 12 mm concentric insertion site roughly 10 mm in depth as a dowel recipient defect. A distal tibia allograft dowel was appropriately measured and prepared at the back table. This was inserted into the recipient site and was flush to the remainder of the glenoid and deemed adequately stable, as depicted in Figure 2. Subscapularis repair using 3 double-loaded suture anchors at the articular margin was then performed prior to closure. We also elected to place 2 figure-of-8 sutures between the upper subscapularis and leading edge of the supraspinatus to serve as an additional buttress. Excellent hemostasis was obtained and standard closure of the deltopectoral approach was completed prior to the patient awakening without any complications.

Postoperative protocol included sling immobilization for 4 weeks at which point she began range of motion progressing from passive to active assisted while limiting external rotation to 45° until 12 weeks postoperatively. Strengthening protocol during this time included rotator cuff, deltoid, and scapular stabilizer focused isometric exercises beginning at 6 weeks postoperatively and progressing to bands to weights until 12 weeks with a 5 lb weight limit. After 12 weeks, she progressed to no motion restrictions while continuing strengthening with eccentrics, plyometrics, and sport-specific exercises beginning 4 months postoperatively. Full return to competitive play was allowed 6 months postoperatively.

**Figure 3:**
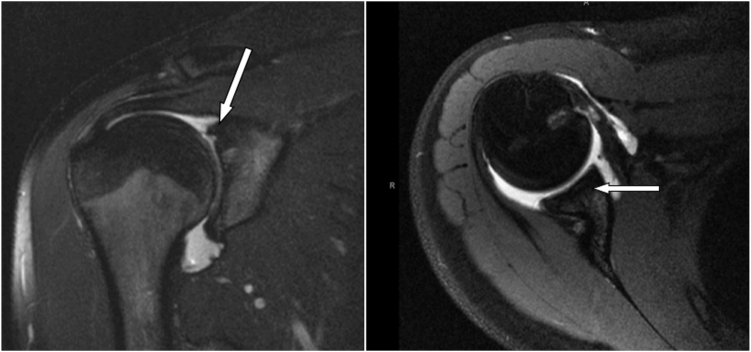
One-year postoperative coronal (*left*) and axial (*right*) T2-weighted MRI of right shoulder demonstrating healed subscapularis and incorporation of glenoid graft at the anterosuperior margin of the glenoid. *MRI*, magnetic resonance image.

The patient’s right shoulder pain completely resolved. Of note, she did sustain an axillary and musculocutaneous nerve palsy postoperatively that resolved after 6 months. At her one-year follow-up visit, the patient reported excellent function without any right shoulder complaints. MRI obtained at that time revealed a completely healed subscapularis and complete incorporation of the glenoid OCA and appropriate, as well as excellent deltoid bulk as depicted in Figure 3. At her two-year follow-up, she reported a Subjective Shoulder Score of 100, a visual analog scale pain score of 0, and an American Shoulder and Elbow Surgeons shoulder score of 100. She reported that she was completely satisfied with the outcome of her surgery and had not had any further reoperations. She returned to gymnastics without limitations and is performing at an elite level at this time without any right shoulder pain or weakness.

## Discussion

OCDs of the glenoid have variable presentations and can cause significant dysfunction in the young and active population. Shoulder pathology in this demographic provides a treatment dilemma as the goal is to remain as conservative as possible while still seeking return to prior level of activity and preventing further surgical procedures. After discerning the cause of a patient’s shoulder pain, OCDs of the glenoid typically undergo nonoperative treatment with a focus on activity modification followed by shoulder strengthening, periscapular stabilization, and rotator cuff strengthening.^24^ In review of these conservative measures applied to lesions of the knee, complete activity restriction alone sustained for 6 months yielded clinical or radiologic healing rates approaching 81 to 96%.^1^ However, it has been reported that these lesions may progress to surgical treatment up to 35% of the time in the pediatric population if they include unstable appearance on magnetic resonance imaging, presence of loose bodies, and failure to heal with nonsurgical management.^23^ Beyond nonoperative treatment, there is not a clearly defined treatment algorithm that exists for OCDs of the glenoid as the literature is sparse in this regard.^7^^,^^11^^,^^22^, ^23^, ^24^ This case report provides a successful example of a treatment pathway for OCD of the glenoid.

The bulk of existing evidence regarding surgical treatment approaches for OCD lesions primarily originates from studies conducted on the knee, elbow, and ankle. The most frequently documented techniques include microfracture, autologous chondrocyte implantation, as well as osteochondral autografts and allografts.

Microfracture, a form of marrow stimulation therapy, consists of debriding an existing chondral defect and creating small holes in the subchondral bone, allowing marrow elements to enter the chondral defect and promote new fibrocartilage deposition.^22^ In young patients undergoing microfracture surgery for glenohumeral chondral defects, clinical failure rates have been shown to approach 33 to 42%.^22^ In the case of this patient, she did not experience adequate relief of symptoms from her initial surgery employing this technique, likely to due to the larger size of the lesion which has shown to be correlated with poorer outcomes.^12^

The technique of autologous chondrocyte implantation or matrix induced autologous chondrocyte implantation involves a two-stage procedure consisting of initial arthroscopy with chondrocyte biopsy, chondrocyte culture, and subsequent open procedure for chondrocyte implantation.^8^ Limited case reports involving the glenohumeral joint have demonstrated satisfactory results using these techniques.^2^^,^^3^^,^^20^

Osteochondral implantation can be performed using an autograft or allograft, and the success of this technique has been attributed to the presence of viable chondrocytes, growth factors, and extracellular matrix proteins to assist with cartilage repair. Successful reports of autograft implantation for chondral defects of the glenoid have been described by Wyland, Junker, and Tsujino.^9^^,^^21^^,^^24^ The advantage of using this technique with an allograft is that it can be performed without donor site morbidity while still being a single-stage procedure as opposed to autologous chondrocyte implantation.^14^ Studies investigating outcomes for OCD lesions in the knee treated by OCA transplantation noted a 93% graft survivorship at 10 years with 95% patient satisfaction.^19^ Similar success has been shown in the pediatric population with 10-year graft survivorship reported at 90% with a patient satisfaction rate of 89%.^15^

As compared with the knee, there is relatively scarce literature on the use of OCA transplantation for OCD lesions in the upper extremity. Mirzayan et al describe a series of young baseball athletes with OCD lesions of the capitellum treated successfully with OCA transplantation.^13^ In a study performed by Riff et al, OCA transplantation of humeral head chondral defects yielded satisfactory outcomes in 55% of patients.^18^

Given the high rates of success seen in the available literature, the authors of this report elected to use an OCA from the distal tibia to repair the glenoid OCD lesion in this patient. A distal tibial allograft was selected after successful reports by Provencher et al who suggest that the dense trabecular subchondral bone of the distal tibia provides robust initial stability along with a well-matched radius of curvature between the humeral head and lateral articular surface of the distal tibia.^16^ For these reasons along with limited access to uncontaminated glenoid allografts, a distal tibial allograft was chosen for osteochondral transplantation in this patient. The use of the standard deltopectoral approach for this surgery requires care, particularly when retracting the deltoid so as not to injure the axillary nerve. However, in our case, the patient fully recovered from an initial axillary nerve palsy.

Of the available literature describing surgical treatment of OCD lesions in the glenoid, this case report is the first to our knowledge to describe the technique of OCA transplantation to the glenoid for a superocentral OCD using a distal tibia allograft with a successful one-year postoperative outcome in a pediatric patient. This report presents evidence of exceptional clinical and radiographic outcomes 2 years after surgery, further supplementing the limited surgical management options available for this condition.

## Conclusion

As demonstrated in this case report, distal tibial allograft transplantation for the treatment of an OCD of the glenoid can be used to achieve satisfactory outcomes.

## Disclaimers:

Funding: No funding was disclosed by the authors.

Conflicts of interest: Travis Maak is a paid consultant for and speaker for Arthrex; participates in fellowship support for Smith & Nephew; and is on the CORR editorial board. Peter Chalmers is a paid consultant for Depuy-Mitek, Exactech, DJ Orthopaedics, and Smith & Nephew; received intellectual property royalties from Depuy, Exactech, and Responsive Arthroscopy; receives publishing royalties from the *Journal of Shoulder and Elbow Surgery*, and has stock in TitinKM Biomedical. The other authors, their immediate families, and any research foundation with which they are affiliated have not received any financial payments or other benefits from any commercial entity related to the subject of this article.

Patient consent: Obtained.

## References

[1] Andriolo L., Candrian C., Papio T., Cavicchioli A., Perdisa F., Filardo G. (2019). Osteochondritis dissecans of the knee - conservative treatment strategies: a systematic review. Cartilage.

[2] Boehm E., Minkus M., Scheibel M. (2020). Autologous chondrocyte implantation for treatment of focal articular cartilage defects of the humeral head. J Shoulder Elbow Surg.

[3] Buchmann S., Salzmann G.M., Glanzmann M.C., Wörtler K., Vogt S., Imhoff A.B. (2012). Early clinical and structural results after autologous chondrocyte transplantation at the glenohumeral joint. J Shoulder Elbow Surg.

[4] Camp C.L., Barlow J.D., Krych A.J. (2015). Transplantation of a tibial osteochondral allograft to restore a large glenoid osteochondral defect. Orthopedics.

[5] Fiegen A., Leland D.P., Bernard C.D., Krych A.J., Barlow J.D., Dahm D.L. (2021). Articular cartilage defects of the glenohumeral joint: a systematic review of treatment options and outcomes. Cartilage.

[6] Ghahremani S., Griggs R., Hall T., Motamedi K., Boechat M.I. (2014). Osteochondral lesions in pediatric and adolescent patients. Semin Musculoskelet Radiol.

[7] Gross C.E., Chalmers P.N., Chahal J., Van Thiel G., Bach B.R., Cole B.J. (2012). Operative treatment of chondral defects in the glenohumeral joint. Arthroscopy.

[8] Hinckel B.B., Gomoll A.H. (2017). Autologous chondrocytes and next-generation matrix-based autologous chondrocyte implantation. Clin Sports Med.

[9] Junker M., Kircher J. (2020). Reconstruction of a central full-thickness glenoid defect using osteochondral autograft technique from the ipsilateral knee. Indian J Orthop.

[10] Kim S.-H., Noh K.-C., Park J.-S., Ryu B.-D., Oh I. (2005). Loss of chondrolabral containment of the glenohumeral joint in atraumatic posteroinferior multidirectional instability. J Bone Joint Surg Am.

[11] Logli A.L., Leland D.P., Bernard C.D., Sanchez-Sotelo J., Morrey M.E., O’Driscoll S.W. (2020). Capitellar osteochondritis dissecans lesions of the elbow: a systematic review of osteochondral graft reconstruction options. Arthroscopy.

[12] Millett P.J., Huffard B.H., Horan M.P., Hawkins R.J., Steadman J.R. (2009). Outcomes of full-thickness articular cartilage injuries of the shoulder treated with microfracture. Arthroscopy.

[13] Mirzayan R., Lim M.J. (2016). Fresh osteochondral allograft transplantation for osteochondritis dissecans of the capitellum in baseball players. J Shoulder Elbow Surg.

[14] Mirzayan R., Sherman B., Chahla J. (2018). Cryopreserved, viable osteochondral allograft for the treatment of a full-thickness cartilage defect of the glenoid. Arthrosc Tech.

[15] Murphy R.T., Pennock A.T., Bugbee W.D. (2014). Osteochondral allograft transplantation of the knee in the pediatric and adolescent population. Am J Sports Med.

[16] Provencher M.T., LeClere L.E., Romeo A.A. (2008). Subpectoral biceps tenodesis. Sports Med Arthrosc Rev.

[17] Ramirez M.A., Ramirez J.M., Murthi A.M. (2015). Arthroscopic management of a glenohumeral osteochondral defect using particulated juvenile cartilage allograft: a case report. JBJS Case Connect.

[18] Riff A.J., Yanke A.B., Shin J.J., Romeo A.A., Cole B.J. (2017). Midterm results of osteochondral allograft transplantation to the humeral head. J Shoulder Elbow Surg.

[19] Sadr K.N., Pulido P.A., McCauley J.C., Bugbee W.D. (2016). Osteochondral allograft transplantation in patients with osteochondritis dissecans of the knee. Am J Sports Med.

[20] Steinmetz G., Hamilton J., Fernandes C., Bond J. (2020). Matrix-induced autologous chondrycte implantation for a glenoid chondral defect: a case report. JBJS Case Connect.

[21] Tsujino S., Tsujino M., Tsujino A. (2018). Unstable osteochondritis dissecans of the glenoid fixed with autogenous osteochondral plugs in a college baseball player: a case report. JBJS Case Connect.

[22] Wang K.C., Frank R.M., Cotter E.J., Davey A., Meyer M.A., Hannon C.P. (2018). Long-term clinical outcomes after microfracture of the glenohumeral joint: average 10-year follow-up. Am J Sports Med.

[23] Weiss J.M., Nikizad H., Shea K.G., Gyurdzhyan S., Jacobs J.C., Cannamela P.C. (2016). The incidence of surgery in osteochondritis dissecans in children and adolescents. Orthop J Sports Med.

[24] Wyland D.J., Beicker C. (2016). Osteochondral autograft transfer technique for glenoid osteochondral defect. Arthrosc Tech.

